# Real-time digital dermatitis detection in dairy cows on Android and iOS apps using computer vision techniques

**DOI:** 10.1093/tas/txae168

**Published:** 2025-02-05

**Authors:** Agam Dwivedi, Marlee Henige, Kelly Anklam, Dörte Döpfer

**Affiliations:** School of Veterinary Medicine, University of Wisconsin-Madison, Madison, WI 53706, USA; School of Veterinary Medicine, University of Wisconsin-Madison, Madison, WI 53706, USA; School of Veterinary Medicine, University of Wisconsin-Madison, Madison, WI 53706, USA; School of Veterinary Medicine, University of Wisconsin-Madison, Madison, WI 53706, USA

**Keywords:** animal welfare, app deployment, cattle, computer vision, deep learning, YOLO

## Abstract

The aim of the study was to deploy computer vision models for real-time detection of digital dermatitis (DD) lesions in cows using Android or iOS mobile applications. Early detection of DD lesions in dairy cows is crucial for prompt treatment and animal welfare. Android and iOS apps could facilitate routine and early DD detection in cows’ feet on dairy and beef farms. Upon detecting signs of DD, dairy farmers could implement preventive and treatment methods, including foot baths, topical treatment, hoof trimming, or quarantining cows affected by DD to prevent its spread. We applied transfer-learning to DD image data for 5 lesion classes, M0, M4H, M2, M2P, and M4P, on pretrained YOLOv5 model architecture using COCO-128 pretrained weights. The combination of localization loss, classification loss, and objectness loss was used for the optimization of prediction performance. The custom DD detection model was trained on 363 images of size 416 × 416 pixels and tested on 46 images. During model training, data were augmented to increase model robustness in different environments. The model was converted into TFLite format for Android devices and CoreML format for iOS devices. Techniques such as quantization were implemented to improve inference speed in real-world settings. The DD models achieved a mean average precision (mAP) of 0.95 on the test dataset. When tested in real-time, iOS devices resulted in Cohen’s kappa value of 0.57 (95% CI: 0.49 to 0.65) averaged across the 5 lesion classes denoting the moderate agreement of the model detection with human investigators. The Android device resulted in a Cohen’s kappa value of 0.38 (95% CI: 0.29 to 0.47) denoting fair agreement between model and investigator. Combining M2 and M2P classes and M4H and M4P classes resulted in a Cohen’s kappa value of 0.65 (95% CI: 0.54 to 0.76) and 0.46 (95% CI: 0.35 to 0.57), for Android and iOS devices, respectively. For the 2-class model (lesion vs. non-lesion), a Cohen’s kappa value of 0.74 (95% CI: 0.63 to 0.85) and 0.65 (95% CI: 0.52 to 0.78) was achieved for iOS and Android devices, respectively. iOS achieved a good inference time of 20 ms, compared to 57 ms on Android. Additionally, we deployed models on Ultralytics iOS and Android apps giving kappa scores of 0.56 (95% CI: 0.48 to 0.64) and 0.46 (95% CI: 0.37 to 0.55), respectively. Our custom iOS app surpassed the Ultralytics apps in terms of kappa score and confidence score.

## Teaser

Custom iOS apps were the better edge devices for real-time digital dermatitis (DD) detection as compared to custom Android and the Ultralytics apps. iOS apps performed well in recognizing DD lesions in real-time in dairy farms and therefore can be used for early detection of DD.

## Introduction

Digital dermatitis (DD) is characterized by the formation of ulcerative lesions on the skin of the feet of cattle (Klitgaard et al., 2014). The primary cause of this infection is Treponema bacteria which invade the skin of the cow’s feet leading to the inflammation and the formation of painful lesions. Digital dermatitis induces lameness and distress in affected animals (Palmer and O’Connell, 2015). Reports indicate that 10% to 40% of lameness cases in dairy herds can be attributed specifically to DD (Wilson-Welder et al., 2015). The lameness results in diminished milk yield, decreased fertility rates, and financial losses for farmers due to the drop in productivity, treatment expenses, and the management cost associated with this condition.

The progression of DD lesions can be divided into 6 distinct stages. The first stage M0, represents the initial phase where the cow feet are in a healthy state, devoid of any lesions. The M1 stage is the early stage where the lesions are still small (<2 cm). The active, ulcerative stage, M2, involves the appearance of a red lesion >2 cm in diameter located between the claw’s heels. As the condition advances, the M2P stage occurs where the M2 lesions are encircled by proliferative epithelial tissue. In the healing phase M3, the M2 lesions are covered by a non-painful scab. In instances where the M2 lesions fail to heal, a chronic stage of DD can develop, described in 2 different stages. i) M4H is characterized by the thickening of the epithelial tissue (hyperkeratosis) and ii) M4P involves the proliferative growth of epithelial tissue (heel warts).

Detecting DD involves observing the feet of cows. Numerous techniques can be employed for this purpose, including visual inspection, mobility assessment, and monitoring of the overall health of the herd. The most common detection method is visual inspection, which requires regular examination of the feet to identify early signs of DD. Visual inspection for DD can be performed on lifted feet in a foot-trimming chute, which is considered the gold standard but is labor-intensive. Alternatively, it can be done in the parlor using a mirror or during pen walks, which are less labor-intensive but potentially less sensitive. This approach is neither automated nor standardized, opening the opportunity for applied computer vision (CV) and object detection in real-time, requiring the development of a more efficient solution for detecting DD.

Computer vision (What is Computer Vision? | IBM, no date) is a field of artificial intelligence focused on enabling computers to interpret and understand visual information from the world. Computer vision has seen significant advancements and applications in various research areas such as X-ray analysis, brain tumor detection, and dermatological disease detection. Computer vision techniques can help in the detection, monitoring, and management of DD in cattle. Object detection in DD involves identifying and localizing lesions within images, videos, and livestreams. Object detection employs 2 methods: single-stage and 2-stage detections. In the single-stage approach, object bounding boxes and classes are predicted directly, whereas the 2-stage approach first proposes potential regions before classification and refinement of detection. The CV models such as You Only Look Once (YOLO) (Redmon et al., 2016), Single Shot Detector (SSD) (Liu et al., 2016), and Faster Region-based Convolutional Neural Network (RCNN) (Ren et al., 2016) are a few state-of-the-art architectures for object detection. Faster RCNN uses a 2-stage approach, making it more accurate but with a longer inference time, i.e., the time between detection and classification. YOLOv5 (Jocher, 2020) and SSD employ one-stage algorithms for object detection with slightly reduced accuracy but higher real-time performance. Studies indicate that YOLOv5 shows better accuracy in identifying smaller objects within images in comparison to Faster RCNN.

The CV models can be deployed in different forms in dairy farms. There are various devices that can be used such as microcontrollers, Android apps, and iOS devices. The challenge of deploying microcontrollers in the field lies in the limited internet connectivity, especially in remote dairy farms. Transmitting video feeds from the microcontrollers to the cloud for analysis by veterinarians becomes difficult due to this connectivity issue. Given this challenge of limited internet connectivity, edge devices such as Android and iOS smartphones offer a practical solution for model deployment and inference in real time. CV models can be deployed directly on the smartphone app, allowing farmers to analyze cows locally without relying on cloud services. Smartphones are convenient and can be regularly used on dairy farms, providing farmers with a practical tool for DD detection using CV.

The aim of the study is to deploy CV models for real-time detection of DD lesions in cattle using Android or iOS applications. Early detection of DD lesions in dairy cows is crucial for prompt treatment and animal welfare. Android and iOS apps can facilitate routine checks of cow feet on dairy farms. Upon detecting symptoms of DD, dairy farmers can implement treatment methods, including foot baths, antibiotics, hoof trimming, or quarantining cows affected by DD to prevent its spread. Administering early treatment decreases the severity of the condition but also enhances overall productivity (Schulz et al., 2016).

## Materials and Methods

The study started with acquiring, analyzing, and pre-processing the data suitable for CV models. Using the PyTorch framework (PyTorch, no date) in Python, the dataset is trained using YOLOv5s (Jocher, 2020) architecture. Multiple train-validation splits are created, each used to independently train the model to determine the best model. Evaluation of these models is based on mean average precision (mAP), inference speed, and validation loss. Qualitative analysis is done to assess the accuracy of the CV model’s bounding boxes around cow feet. The PyTorch model is converted to a TensorFlow Lite or CoreML model for its deployment on an Android or an iOS device to detect in real time.

The app functions without requiring internet access, making it valuable in remote dairy farms with limited or no internet connectivity.

### Data Collection

Data was gathered from one midwest dairy farm. There are different ways to position camera modules across various sections of the farms, such as milking carousels, milking parlors, and free-stall barns. An alternative method for collecting data about hoof health is checking cow’s hind feet while cows are feeding in headgates. After the data collection process, videos in MP4 format were assessed and images were generated from the video. Images were checked for blurriness and quality, capturing one image (JPG or PNG) per second of the video. These images were then resized to 416 × 416 pixels and corresponding annotation files in XML format were created. The XML files contain the file names, labels, bounding boxes, and the box’s coordinates. The next step was to convert the annotation file to a suitable YOLO (Redmon et al., 2016) format.

The development of DD lesions can be categorized into 6 stages—M0, M1, M2, M2P, M4H, and M4P. In our research, we are excluding M1 lesions from consideration because these are not consistently visible in the images required to train the model. The visibility issues can be attributed to the fact that the images are taken of standing cow feet in the milking parlor. We have 240, 17, 51, 114, and 108 instances of each of the M0, M2, M2P, M4H, and M4P classes respectively. The ground truth images are scored by a single investigator and the scores were recorded in MS Excel v16.68. The investigator is a veterinarian who is an expert in microbiology and predictions of infectious diseases in animals. The flexible image augmentation library Albumentations (Albumentations: fast and flexible image augmentations, no date) was used, among which: Median Blur, Blur, and Grayscale, to effectively increase and diversify the dataset, promoting better class balance.

### Data Configuration

We applied transfer-learning for DD data on pretrained YOLOv5 architecture using COCO-128 pretrained weights (COCO—Common Objects in Context, no date). Transfer-learning is a machine-learning technique where a model trained on one task is repurposed for a related task, often leading to improved performance with less data and training time. We used the combination of localization loss, classification loss, and objectness loss as the loss function. The localization loss measures how accurately the model predicts the bounding box coordinates, typically represented as the top-left and bottom-right corners, of the objects in the image. The classification loss measures the dissimilarity between the predicted class probabilities and the true class labels. The objectness loss penalizes the model when it misclassifies objects or fails to predict their presence. In addition, objectness loss penalizes confidence scores for predicted boxes that do not contain objects. The combinations of these 3 losses were used to train object detection models.

For training optimization, we used Stochastic Gradient Descent (SGD) with a learning rate of 0.01. The SGD used a random subset of data, so-called a batch, to estimate the gradient and update the parameters in the direction that reduces the loss. During the training process, a batch size of 16 with a maximum of 300 epochs and an early stopping mechanism were applied.

### Model Training

For the model training, we created a virtual environment in Pythonv3.8.16 with the PyTorchv2.0.0 framework installed. The training data encompassed 90% of the dataset, while the remaining 10% served for model testing. Our approach used the pretrained YOLOv5 architecture and transfer-learning techniques for the DD data. YOLOv5 (YOLO version 5) is an object detection architecture that employs a single neural network to simultaneously predict object bounding boxes and their classes. It introduces a streamlined architecture with multiple detection head sizes, being one of the components of the network, to detect objects at different scales and allow flexibility in the input dimensions of the images. YOLOv5 uses a lightweight backbone network (CSPDarknet53) (Wang et al., 2019) for feature extraction and incorporates the localization loss, classification loss, and objectness loss for accurate predictions. The input to YOLOv5 is an RGB image of variable size, typically resized to a specific input resolution. The model’s output consists of predicted bounding boxes and associated class probabilities. Each bounding box is represented by its coordinates, typically top-left and bottom-right corners. Additionally, objectness confidence scores indicate the likelihood of each predicted bounding box containing an object.

### Model Performance Evaluation

Multiple performance metrics among which mean average precision, precision, and recall, are used to quantify model performance. The mAP is the mean (average) of the average precision (AP) values across all classes (Mean Average Precision (mAP) Explained: Everything You Need to Know, no date). This measure provides an overall assessment of the model’s performance across multiple classes. The mAP that we considered is mAP-50 which refers to the mean average precision computed at a 50% intersection over union (IoU) threshold, indicating object detection accuracy considering moderate overlap between predicted and ground truth bounding boxes. Precision is a metric that measures the accuracy of a model’s positive predictions. It is calculated as the ratio of true positives (TP) to the sum of TP and false positives. Recall is a measure of a model’s ability to correctly identify all positive instances in the dataset. Recall is the ratio of true positives (TP) to the sum of TP and false negatives (FN). It is also known as “sensitivity” or “true positive rate” (Precision, Recall, no date).

To evaluate the agreement between the investigator and the model prediction, Cohen’s kappa score metric was used to measure the level of agreement between 2 evaluators for categorical classes. It is particularly useful when assessing the magnitude of agreement between human investigators. Kappa score can be categorized as follows—0.01 to 0.20: none to slight agreement; 0.21 to 0.40: fair agreement, 0.41 to 0.60: moderate agreement; 0.61 to 0.80: substantial agreement; and 0.81 to 1.00: almost perfect agreement (McHugh, 2012). A confusion matrix for multiclass classification was used to summarize the model’s performance by showing the counts of TP, true negatives, false positives, and FN across multiple classes or categories. High frequency across the diagonal of the confusion matrix reflects a high agreement between a number of model predictions and true class counts (Narkhede, 2021).

For the aforementioned metrics, we also combined the 5 lesion classes into certain categories. For the first categorization, we combined 5 classes into M0, M2, and M4. The M0 stage represented healthy and recovered feet (a combination of M0 and M4H classes). The M2 stage represented the combination of M2 and M2P classes. The M4 stage represented the M4P classes. For the second categorization, we had 2 classes—M0 and M2. Here M0, was the same as indicated above, a combination of M0 and M4H classes. While the M2 class was the combination of M2, M2P, and M4P classes; indicating the comparison between the lesion and non-lesion classes.

### Model Deployment

After training the model successfully, we obtained the.pt model file and converted it into a suitable format for an Android or an iOS app. This involved converting the.pt file into TFLite (TensorFlow Lite | ML for Mobile and Edge Devices, no date) format for Android and CoreML format (Getting a Core ML Model, no date) for iOS. After conversion, we set up Android Studio and XCode projects, respectively, for real-time object detection. Inference code was written for these devices to ensure seamless real-time performance. A User Interface (UI) was crafted for the app, utilizing the device’s camera for real-time DD detection. This interface was then merged with the inference code to facilitate real-time object detection.

#### Custom iOS deployment

**Figure 1.**
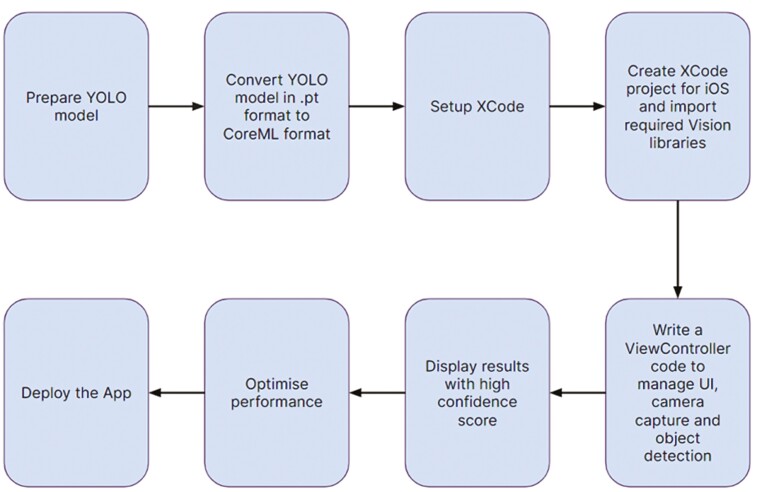
Tasks performed to deploy CoreML model file on a custom iOS app.

CoreML (Getting a Core ML Model, no date) is a machine-learning framework developed by Apple that allows developers to integrate trained machine-learning models into their iOS, macOS, watchOS, and tvOS applications. CoreML supports a variety of tasks, including CV tasks like object detection. The 5 tasks are described below and are illustrated in Figure 1.

1. Model conversion: First, our YOLOv5 model, was trained and converted into the CoreML format. This was achieved using tools like “coremltools” for Python, which convert the model into a CoreML format that can be directly integrated into Apple platforms (Inc, no date).
2. Integration: The converted CoreML model was easily integrated into an iOS app. This involved adding the CoreML model file to the XCode project and then using the CoreML framework to load the model into the app. The code involves writing a ViewController class which has 4 functions—1) Video capture, 2) Video output, 3) Visual layers, and 4) Vision predictions. The Video capture functionality is to find a camera device which in our case is the back camera of the phone. The Video output function is to link the back camera to the display. Visual layer function displays various layers in the real-time video. It includes a preview layer, detection layer, and inference time layer. In vision prediction function, a custom model was loaded and the predictions were computed and displayed using a shape layer and a text layer.
3. Inference: Once the model was loaded into XCode, the app performed object detection on images or video frames. This involved passing the input image through the CoreML model, which generated predictions for object bounding boxes and class probabilities.
4. Postprocessing: After inference, the app performed postprocessing steps to refine the object detection results. This involved filtering out low-confidence predictions, applying non-maximum suppression to remove duplicate detections, and converting bounding box coordinates from normalized values to actual image coordinates.
5. Visual feedback: The app displayed the processed detection results on the UI, by drawing bounding boxes around detected objects and showing class labels.

CoreML simplifies the deployment of machine-learning models on Apple devices and optimizes their performance for real-time inference. This allows developers to create applications that leverage CV capabilities, such as object detection, directly on iOS devices while taking advantage of the hardware acceleration available on these platforms.

#### Custom Android deployment

**Figure 2.**
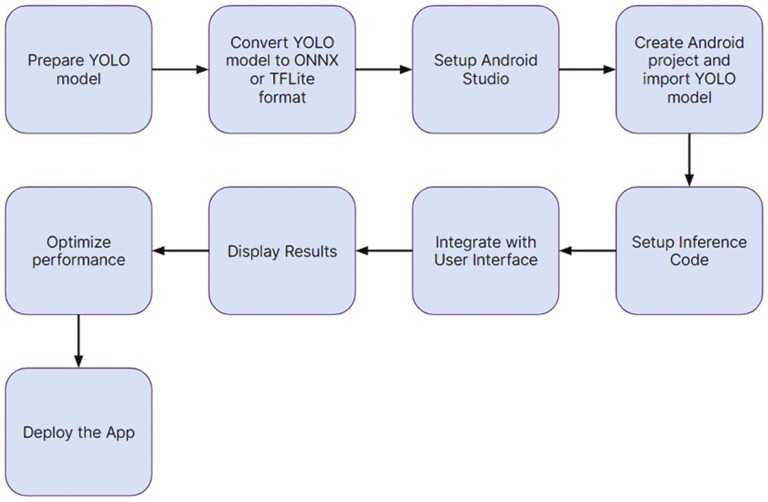
Tasks performed to deploy TFLite model file on a custom Android app.

Deploying a TensorFlow Lite (TFLite) (TensorFlow Lite | ML for Mobile and Edge Devices, no date) model in real-time in Android Studio involved several steps. The tasks are described briefly below and illustrated in Figure 2:

1. Install Android Studio and create an Android project: The initial step involved downloading and installing Android Studio from its official website. After installation, launch Android Studio and either initiate a new Android project or access an existing one. It is essential to have Android Gradle Plugin version 4.2.0 and Gradle version 7.2 installed. Then load the “org.tensorflow.lite” API to utilize the YOLOv5 TFLite model in Android.
2. Add the TFLite model to the project: The TFLite model file (with a “.tflite” extension) was placed in the “*app/src/main/assets*” directory of the Android project. Custom class text file, that will have the classes applicable to our model, was created and placed in the same directory.
3. Initialize TensorFlow Lite Interpreter: In the Android code (e.g., within an Activity or Fragment), the TensorFlow Lite interpreter was initialized to load and use the model. This part was done in the *YOLOv5Classifier.java* file. If there are multiple models, if–else conditions are added to access a particular model and its respective custom classes file. This was done in *DetectorFactory.java* file.
4. Write code for camera module and the UI: The input data were prepared in a format suitable for the model. In our case, input size was 416 × 416 pixels. The camera will take the large-size image captured in real-time and crop it to the aforementioned resolution. The user interface (UI) has the features to test out different models from the list and provide flexibility for choosing Central Processing Unit (CPU) or Graphics Processing Unit (GPU) for real-time object detection.
5. Inference: During the next step, the code was built and the app performed object detection on images or video frames. This involved passing the input image through the TFLite model, which generated predictions for object bounding boxes and class probabilities.
6. Postprocessing and display: After inference, the app applies postprocessing steps to refine the object detection results. This involved filtering out low-confidence predictions, applying non-maximum suppression to remove duplicate detections, and converting bounding box coordinates from normalized values to actual image coordinates. Finally, results were displayed in the Android UI, rendering bounding boxes for object detection and class confidence scores (Confidence Score—an overview | ScienceDirect Topics, no date).

#### Ultralytics Android and iOS deployment

Ultralytics (Ultralytics | Revolutionizing the World of Vision AI, no date) is an open-source software framework and ecosystem designed for CV and deep learning tasks. It is primarily built around the PyTorch deep learning library and aims to simplify and streamline the process of developing, training, and deploying CV models. Ultralytics (UL) provides a range of tools, libraries, and utilities to facilitate various aspects of CV and deep learning projects. For UL deployment, we trained the model using the UL web interface with the same configuration as described in the training process above. After training, the model was exported to CoreML format for an iOS app and TFLite format for an Android app. The Ultralytics HUB app can be installed using the Play Store on Android or App Store on iOS devices. On the app itself, we can select the model that we trained and use it seamlessly in real time.

## Results

The prediction performance of the custom YOLO model applied to the DD data was evaluated based on the mAP score, precision, and recall. Table 1 shows the class-wise values for each of the precision, recall, and mAP metrics as derived from the custom yolov5s.pt model file.

**Table 1.** Precision, Recall, and mAP metrics for 5 DD classes^1^ as derived from the custom yolov5s.pt model file

Classes	Precision	Recall	mAP	No. of test instances
M0	0.937	0.71	0.907	29
M2	0.985	0.996	0.995	1
M2P	0.89	0.994	0.996	3
M4H	0.856	0.848	0.88	14
M4P	0.958	0.998	0.995	11

^1^M0—healthy feet; M2—appeared as a red lesion > 2cm in diameter located between the claw’s heels; M2P—M2 lesions encircled by proliferative epithelial tissue; M4H—characterized by the thickening of the epithelial tissue (hyperkeratosis); M4P—indicated as proliferative growth of epithelial tissue (heel warts).

**Figure 3.**
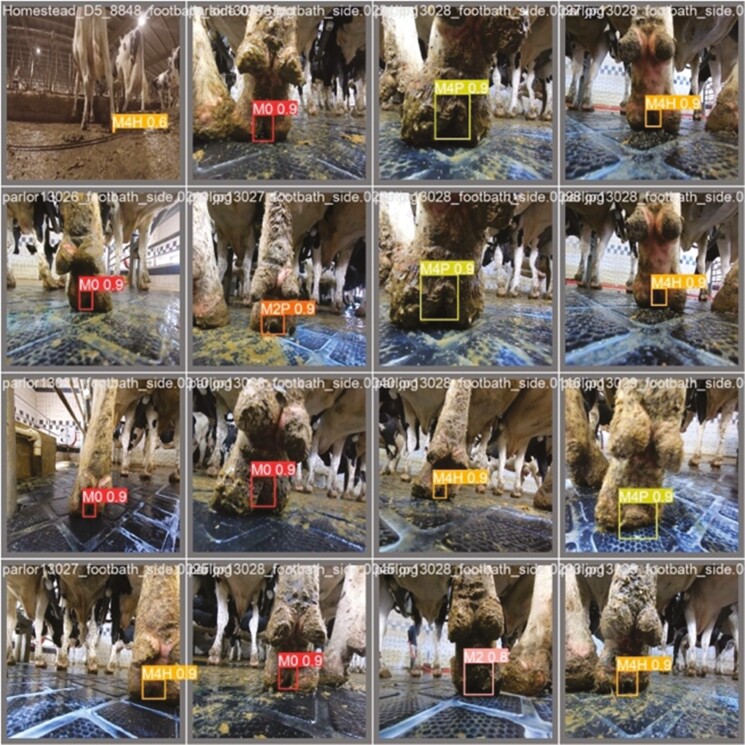
Qualitative analysis with true labels (left) and predicted labels (right) as derived from custom yolov5s.pt model file (train samples = 363, test samples = 46, Epochs = 300). Original and predicted lesions [M0—healthy feet; M2—appeared as a red lesion > 2cm in diameter located between the claw’s heels; M2P—M2 lesions encircled by proliferative epithelial tissue; M4H—characterized by the thickening of the epithelial tissue (hyperkeratosis); M4P—Indicated as proliferative growth of epithelial tissue (heel warts)] are indicated by the bounding boxes.

The qualitative analysis of the results is shown in Figure 3, where we compared the images with true classes with the predicted ones. We found complete agreement between true classes and the predictions. This means all the classes are detected accurately for a given subset of test samples. However, the subset of test samples provided does not represent real-time data from dairy farms. Instead, it serves as a method to verify the model’s performance before deployment in the actual farm.

Cohen’s kappa scores were used to measure the agreement between the annotations provided by an investigator and the predictions made by the CV model on iOS and Android devices. Table 2 illustrates the average kappa scores across the 5 lesion classes with and without the data that cannot be scored. The score of 0.57 (95% CI: 0.49 to 0.65) has a moderate level of agreement between the investigator and the custom iOS app (without “can’t score” classes). This kappa score is higher than the custom Android app, where the agreement between the model and the investigator is 0.38 (95% CI: 0.29 to 0.47). Table 2 also shows kappa scores including the classes that cannot be scored—giving the highest kappa score of 0.55 for the iOS-UL app.

**Table 2.** Cohen’s kappa score for dataset with (*N* = 161) and without (*N* = 109 for custom apps and *N* = 68 for Ultralytics [UL] apps)^1^ classes that cannot be scored by the respective models. Highest kappa score in each row is denoted by bold

	Custom iOS app	Custom Android app	iOS-UL app	Android-UL app
Kappa score without classes which cannot be scored	**0.57**	0.38	0.56	0.46
Kappa score with classes which cannot be scored	0.432	0.43	**0.55**	0.3

^1^ *N*, number of real-time samples for testing DD detection.

Table 3 shows the number of test samples not scored by the investigator and the custom and Ultralytics (iOS and Android) apps respectively. Android-UL app performs the worst as it is unable to detect approximately 58.3% of samples. Custom iOS app surpassed other evaluators with only 5.5% of samples undetected.

**Table 3.** Test samples that cannot be scored by different evaluators. Out of 161 samples, investigator was not able to detect 39 samples, implying bad lighting or dirty cow feet. However, custom iOS app was able to detect 152 samples, making it most robust among all the evaluators. Android Ultralytics (UL) app perform the worst as it is not able to detect even 50% of the samples. Lowest number of samples not detected by a particular app is denoted by bold.

Evaluator	Number of test samples not detected (Total 161 test samples)
Investigator	39 (24%)
Custom iOS app	**9 (5.5%)**
Custom Android app	42 (26%)
iOS-UL app	28 (17%)
Android-UL app	94 (58.38%)

**Figure 4.**
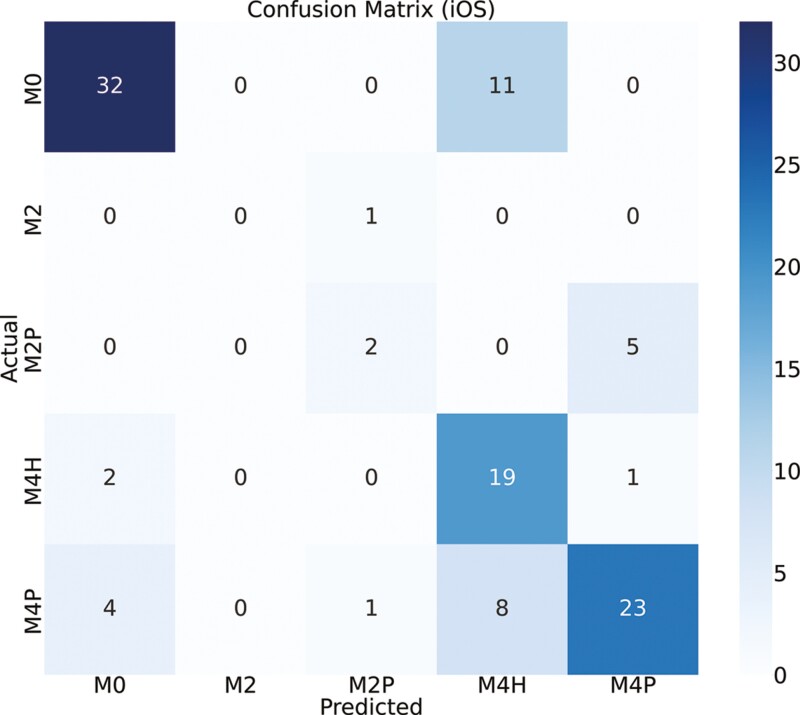
Confusion matrix between actual and predicted class labels [M0—healthy feet; M2—appeared as a red lesion > 2cm in diameter located between the claw’s heels; M2P—M2 lesions encircled by proliferative epithelial tissue; M4H—characterized by the thickening of the epithelial tissue (hyperkeratosis); M4P—Indicated as proliferative growth of epithelial tissue (heel warts)] for custom yolov5s models in iOS app (*N* = 109). Number of samples on the main diagonal of the confusion matrix indicates correctly classified samples. *N*, number of real-time samples for testing DD detection.

**Figure 5.**
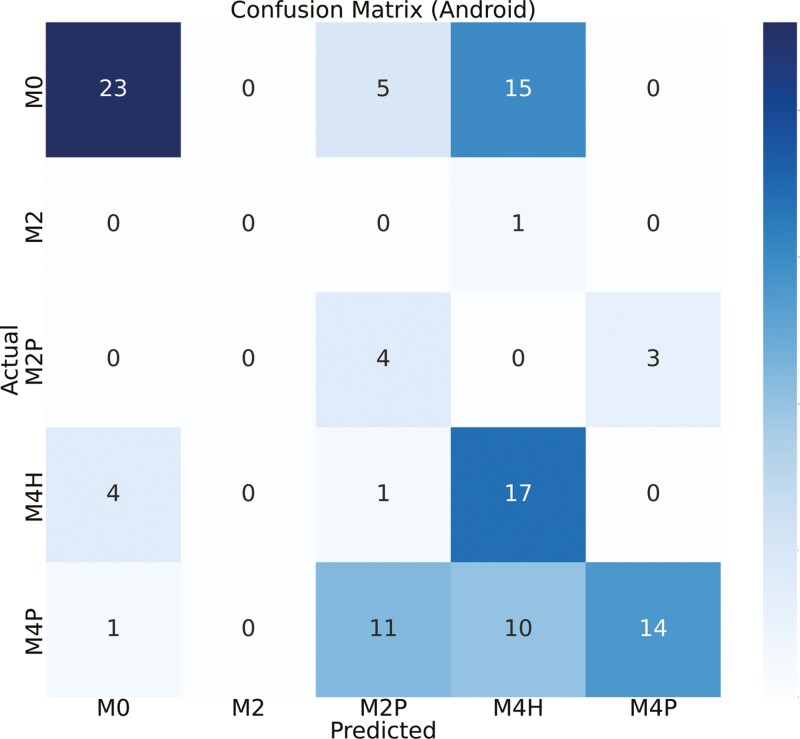
Confusion matrix between actual and predicted class labels [M0—healthy feet; M2—appeared as a red lesion > 2cm in diameter located between the claw’s heels; M2P—M2 lesions encircled by proliferative epithelial tissue; M4H—characterized by the thickening of the epithelial tissue (hyperkeratosis); M4P—Indicated as proliferative growth of epithelial tissue (heel warts)] for custom yolov5s models in Android app (*N* = 109). Number of samples on the main diagonal of the confusion matrix indicates correctly classified samples. *N*, number of real-time samples for testing DD detection.

**Figure 6.**
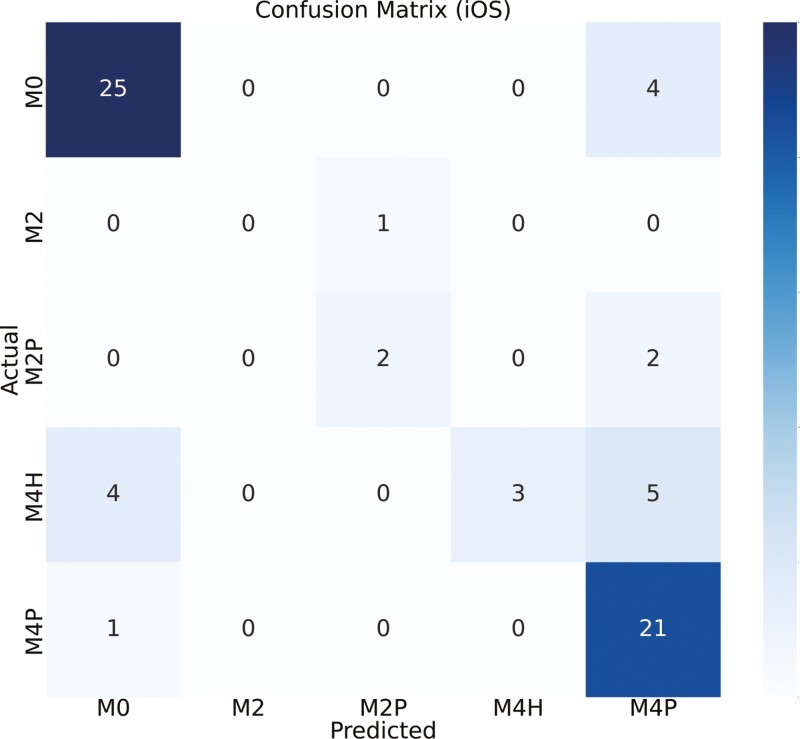
Confusion matrix between actual and predicted class labels [M0—healthy feet; M2—appeared as a red lesion > 2cm in diameter located between the claw’s heels; M2P—M2 lesions encircled by proliferative epithelial tissue; M4H—characterized by the thickening of the epithelial tissue (hyperkeratosis); M4P—Indicated as proliferative growth of epithelial tissue (heel warts)] for Ultralytics yolov5s models in iOS app (*N* = 68). Number of samples on the main diagonal of the confusion matrix indicates correctly classified samples. *N*, number of real-time samples for testing DD detection.

**Figure 7.**
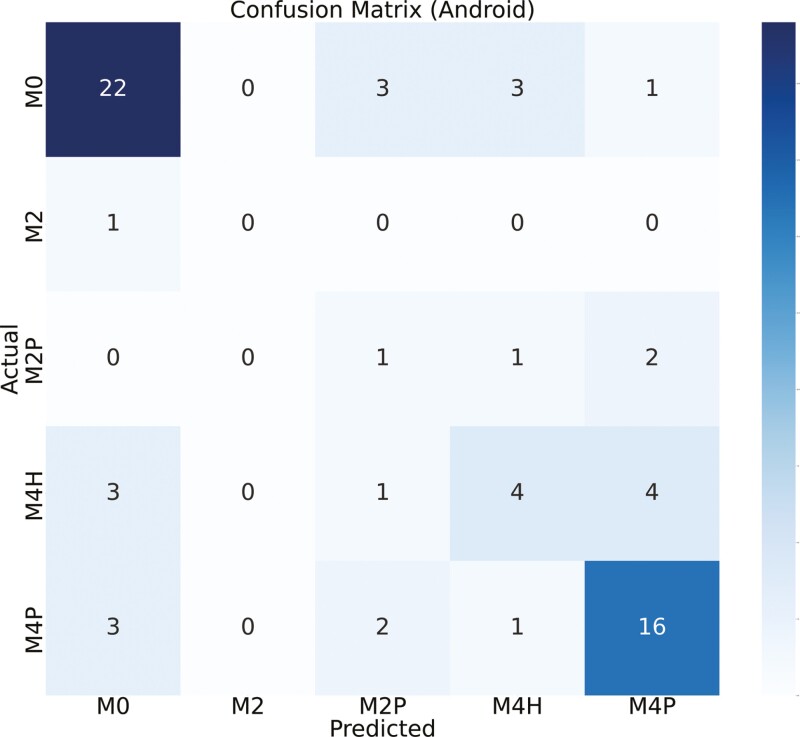
Confusion matrix between actual and predicted class labels [M0—healthy feet; M2—appeared as a red lesion > 2cm in diameter located between the claw’s heels; M2P—M2 lesions encircled by proliferative epithelial tissue; M4H—characterized by the thickening of the epithelial tissue (hyperkeratosis); M4P—Indicated as proliferative growth of epithelial tissue (heel warts)] for Ultralytics yolov5s models in Android app (*N* = 68). Number of samples on the main diagonal of the confusion matrix indicates correctly classified samples. *N*, number of real-time samples for testing DD detection.

Figures 4 and 5 show the confusion matrix between the true labels and the predicted labels using custom iOS and Android apps, respectively. The diagonal of the confusion matrix indicates the number of correct predictions. The color of the confusion matrix indicates the density of the predictions. M0 is detected more accurately than other classes (as seen by the color of the M0 cell). A total of 109 samples were tested in real time. Figures 6 and 7 show the confusion matrix between the true labels and the predicted labels using Ultralytics iOS and Android apps, respectively. A total of 68 samples are used to test in real time. The decrease in number of samples was because there were many samples that were not scored by the Ultralytics apps.

In Table 4, we compare the kappa values of various edge devices—custom iOS and Android apps with Ultralytics iOS and Android apps. We calculate the kappa values for a 5-class, 3-class, and 2-class categories. Custom iOS app excelled than other apps in terms of kappa score for the 3 categories mentioned above.

**Table 4.** Comparison of kappa scores between our custom app and Ultralytics (UL) HUB app. Highest kappa score for each row is denoted by bold.

Kappa Score	Custom iOS (*N* = 109)^1^	Custom Android (*N* = 109)	iOS UL (*N* = 68)	Android UL (*N* = 68)
2-Class model (M0 and M2)^2^	**0.74 (95% CI: 0.63 to 0.85)**	0.65 (95% CI: 0.52 to 0.78)	0.7 (95% CI: 0.59 to 0.81)	0.54 (95% CI: 0.42 to 0.66)
3-Class model (M0, M2 and M4)	**0.65 (95% CI: 0.54 to 0.76)**	0.46 (95% CI: 0.35 to 0.57)	0.63(95% CI: 0.52 to 0.74)	0.48 (95% CI: 0.37 to 0.59)
5-Class model (M0, M2, M2P, M4H, M4P)	**0.57 (95% CI: 0.49 to 0.65)**	0.38 (95% CI: 0.29 to 0.47)	0.56(95% CI: 0.48 to 0.64)	0.46 (95% CI: 0.37 to 0.55)

^1^ *N*, number of real-time samples for testing DD detection.

^2^M0—healthy feet; M2—appeared as a red lesion > 2cm in diameter located between the claw’s heels; M2P—M2 lesions encircled by proliferative epithelial tissue; M4H—characterized by the thickening of the epithelial tissue (hyperkeratosis); M4P—indicated as proliferative growth of epithelial tissue (heel warts).

**Figure 8.**
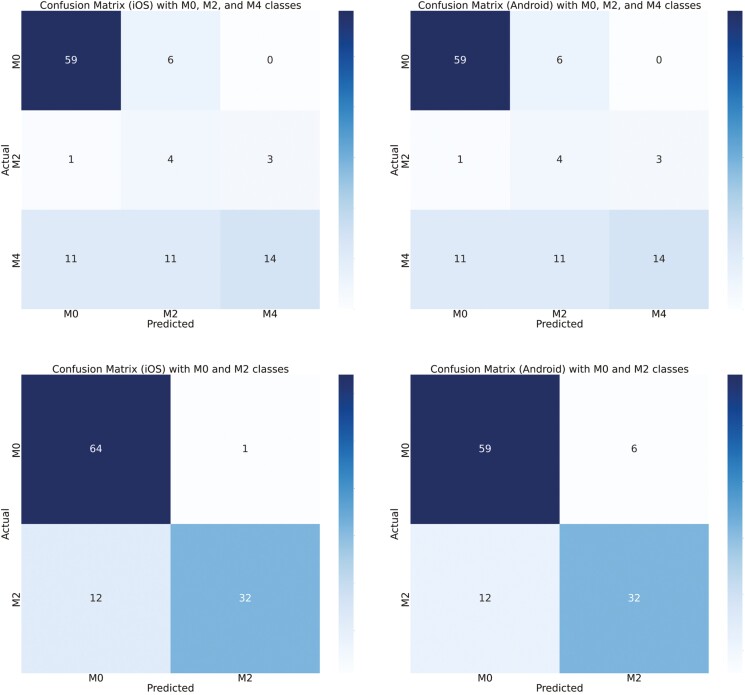
First row compares confusion matrix for custom iOS app and custom Android app detections for 3-class model (M0 => M0 + M4H, M2 => M2 + M2P, M4 => M4P). M0 and M4H are combined, M2 and M2P are combined, and M4P (*N* = 109). Second row compares confusion matrix for custom iOS app and custom Android app detections for 2-class model (M0 => M0 + M4H, M2 => M2 + M2P + M4P). M0 and M4H are combined (denoting healthy or recovered feet) and M2, M2P, and M4P are combined (denoting feet with lesion) (*N* = 109). *N*, number of real-time samples for testing DD detection.

**Figure 9.**
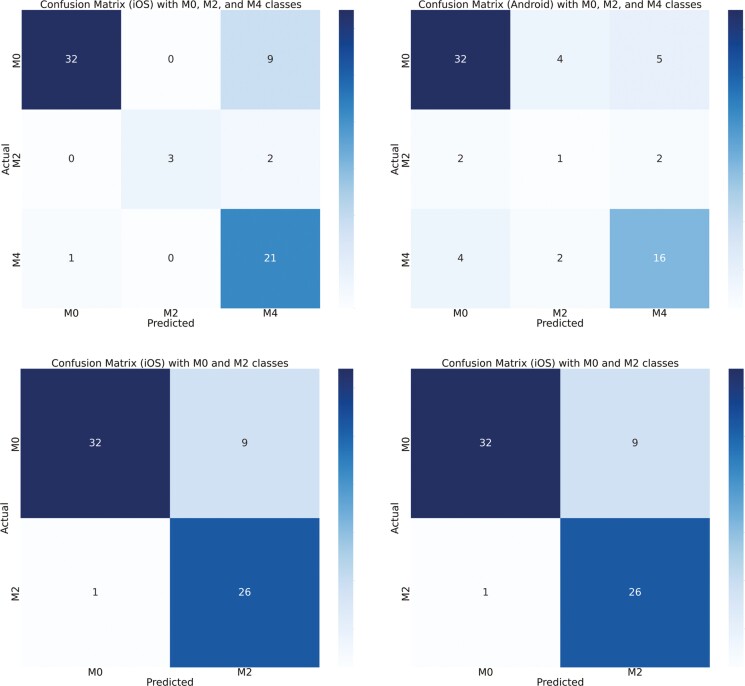
First row compares confusion matrix for iOS-Ultralytics (UL) app and Android-UL app detections for 3-class model (M0 => M0 + M4H, M2 => M2 + M2P, M4 => M4P). M0 and M4H are combined, M2 and M2P are combined, and M4P (*N* = 68). Second row compares confusion matrix for iOS-UL app and Android-UL app detections for 2-class model (M0 => M0 + M4H, M2 => M2 + M2P + M4P). M0 and M4H are combined (denoting healthy or recovered feet) and M2, M2P, and M4P are combined (denoting feet with lesions) (*N* = 68). *N*, number of real-time samples for testing DD detection.

Figure 8 shows the custom iOS and Android app results when we combined the 5 classes into certain categories. The first row shows a confusion matrix where classes are combined into M0, M2, and M4. As we can observe, concentrating the classes together increases the correctness of the predictions. The second row has the confusion matrix with 2 classes—M0 and M2. Figure 9 shows similar results for the Ultralytics iOS and Android apps.

Table 5 displays the average inference time comparison between custom and Ultralytics iOS and Android apps. Inference time refers to the time it takes for a model to process an image frame and produce a detection result. It is a measure of how quickly the model can make predictions once it has been trained. The iOS device performed better than Android with an average inference time of 20 ms. The average inference time of UL Android apps was 142.8 ms, making it difficult for the user to understand the predictions. Table 6 shows that detection made by the custom iOS app has a high confidence score with an average of over 93% across all the classes. Ultralytics iOS app does good as well with an average confidence score of 91% across all the classes.

**Table 5.** Average inference time on different edge devices (*N* = 109 for custom apps and *N* = 68 for Ultralytics (UL) apps)^1^. Highest average inference time among all apps is denoted by bold.

Edge Device	Average inference time (ms)
iOS	**20**
Android	55
iOS UL	33.33
Android UL	142.85

^1^ *N*, number of real-time samples for testing DD detection.

**Table 6.** Average confidence scores for different classes on the custom and Ultralytics Android and iOS apps (*N* = 109 for custom apps and *N* = 68 for Ultralytics apps)^1^. Highest confidence score in each row is denoted by bold.

	Confidence score (Android; %)	Confidence score (iOS; %)	Confidence score (Android Ultralytics; %)	Confidence score (iOS Ultralytics; %)
M0^1^	54.25	**96.58**	34.36	91.63
M2	48	**94.5**	35.2	89.2
M2P	58.6	**93.8**	21.52	90.45
M4H	59.3	**96.9**	22.72	91.33
M4P	50.2	**96.8**	32.22	93.67

^1^ *N*, number of real-time samples for testing DD detection.

^2^M0—healthy feet; M2—appeared as a red lesion > 2cm in diameter located between the claw’s heels; M2P—M2 lesions encircled by proliferative epithelial tissue; M4H—characterized by the thickening of the epithelial tissue (hyperkeratosis); M4P—indicated as proliferative growth of epithelial tissue (heel warts).

## Discussion

Starting with a statistical analysis of the DD data as described in the Results section, we found that the M0 class exhibited the highest frequency, indicating numerous healthy cows in the barn. The M2 class had notably lower representation, with only 16 instances compared to other classes resulting in an imbalance. This suggested the possibility that the cows are in good health or that M2 converted into other lesions overtime. Addressing the dataset’s imbalance was imperative before model training. To tackle this challenge, we employed data augmentation techniques (Albumentations: fast and flexible image augmentations, no date). For the current training setup, we used smoothing techniques like Blur and Median Blur, Color to Grayscale conversion for data augmentation. In the future, we are planning to use augmentation techniques like Contrast Limited Adaptive Histogram Equalization (CLAHE) (OpenCV: cv::CLAHE Class Reference, no date), Brightness and Contrast adjustments.

As observed in Table 1, the highest mAP (Mean Average Precision (mAP) Explained: Everything You Need to Know, no date) scores of 0.995 and 0.996 were achieved for M2 and M2P, respectively, which can be attributed to the fact that there were relatively few instances of M2 and M2P classes. Considering the substantial frequency of test samples in the M0, M4H, and M4P classes, we can conclude that the DD model performed better in those cases. Figure 3 shows that all the classes were predicted correctly with greater than 0.6 confidence score. We infer that our model performance is acceptable because it predicts test data with high confidence. It would be advisable to collect more M2 and M2P lesions to improve model prediction performance. These results look too good to be true because all the predictions are correct on the test dataset. It can be attributed to the fact that all the train and test images are taken in the same environment conditions, which lead to a similarity pattern that is easier to detect by the machine-learning model.

To evaluate the model performance, external validation is essential in real time. The real-time detection of live-feeds from on-site cameras is preferred over images presented to the model because of varying lighting conditions, distances from cow feet, and other factors. To validate the performance from live-feeds to the cameras in real-time, we obtained the Cohen’s kappa scores shown in Table 4. The custom iOS device stood out with regards to the kappa score metric by detecting images and videos under challenging lighting conditions and identifying well from various angles and distances from the cow’s hind feet. For all the 3-categories, custom iOS app performs better than other apps. UL iOS app gives kappa score of 0.56, only slightly less than the custom iOS app kappa score of 0.57 for 5-class model. UL Android app performs worse than iOS apps, but scores a higher kappa value of 0.46 than the custom Android app. Kappa score increases from 5-class to 3-class to 2-class model for all the apps, which means that concentrating classes into applicable categories improves the detections.

As demonstrated in Table 3, the custom iOS app successfully detected 152 out of 161 samples, surpassing even the human annotator who detected 123 samples. In contrast, custom Android detected 119 samples out of 161. UL iOS app detected 83% of the samples as compared to 41.6% of samples in UL Android app. We tried playing with various values of IoU threshold and confidence threshold on UL apps to see why it is not detecting DD properly but still did not see any improvements in detections. One of the tasks in the future could be to analyze the UL code. We analyzed the classes that were not scored by the annotator or the edge devices. As indicated in Table 2, both custom Android and custom iOS app had kappa scores of approximately 0.43 including the predictions which are not scored. The decrease in the kappa score of the custom iOS app may be attributed to its ability to detect challenging samples in real time. For the UL iOS app, kappa score of 0.55 is achieved with classes which cannot be scored. It is because there are so many classes that are not scored, giving a strong correlation between “not scored” classes between the investigator and the UL iOS app.

We observed that the iOS model works better than the Android model. The custom iOS app beats the custom Android app in inference time (Table 5), 20 ms inference time as compared to 55 ms inference time on the custom Android application. UL iOS app predicts with average inference time of 33.33 ms. Android-UL app performs worst of all, with 142.85 ms of average inference time—evaluating only 7 frames/s. The only reason we can think of why Android-UL app is not working well because it is not optimized. Therefore, UL Android app is not serviceable in real-time DD lesion detections with such low fps. The custom iOS app predicts DD lesions in real-time with very high-class confidence averaging around 0.94, while the custom Android app had average predictions for class confidences of 0.54 as shown in Table 6. Similarly, UL iOS app predicts with a high confidence score of 0.91 and UL Android app predicts with an average confidence score of 0.29. Due to such low confidence in UL Android app, it is not reliable and may not be suitable for real-time use. CoreML’s performance speed over TFLite for object detection is due to its optimized alignment with the Apple ecosystem (Reddy, 2021). Models are fine-tuned for Apple device architecture, while hardware acceleration such as the Neural Engine boosts CoreML’s inference speed. Deep integration of the CoreML models with iOS frameworks minimizes overhead computing expenditures during execution. Ongoing backend enhancements, such as quantization, sustain CoreML’s advantage for object detection speed on iOS devices.

The confusion matrix, as depicted in Figure 4, showed the relationship between true labels and the custom iOS app predictions. Notably, 11 out of 43 samples from the M0 class were erroneously detected as M4H, likely due to the visual similarity between M0 and M4H in real time. Conversely, the custom Android app showed poor performance in this scenario, with 5 M0 samples predicted as M2P and 15 as M4H (Figure 5). One sample of M2 is predicted incorrectly by both devices since M2 samples were represented less in the training data set compared to other classes. For the M4H class, both custom iOS and custom Android performed reasonably well, correctly predicting 19 and 17 samples, out of 23. In the case of M2P, Android correctly identified 4 out of 7 samples, while iOS detected 2 out of 7 correctly. For the M4P class detection, custom iOS had higher performance compared to Android, with 23 out of 36 samples correctly identified, whereas Android managed 14 out of 36. The diagonal of the confusion matrix gets more populated as we move from 5-class to 3-class and then to 2-class model which means there is a better agreement. And we could verify this by looking at the kappa score (Table 4). We did not need to always have 5-stage highly accurate model. Sometimes, the requirement was to predict whether there is a DD lesion or not. In this case, we could use our 2-class model. Figure 6 showed the confusion matrix between true labels and UL iOS app predictions. One notable thing was the number of samples in the confusion matrix (*N* = 68). It is because out of 161 samples, only 68 samples are scored by all the investigators, UL iOS app and UL Android app. Most of the M0 and M4P classes were detected by UL iOS application. However, only 3 out of 12 M4H classes were detected accurately by UL iOS application. In detecting M4H, our custom iOS model performed better than UL iOS app. A similar resemblance could be seen in the UL Android confusion matrix (Figure 7), where most of the M0 and M4P are detected, but only 4 out of 12 M4H classes are detected correctly. Similar to custom iOS and Android apps, little to no resemblance was seen in the UL iOS and Android apps for M2 and M2P classes. Again, it was because there are very few samples of M2 and M2P classes in the training and the test set.

Our study marks the initial steps toward deploying CV models for real-time DD detection. However, it has some limitations. Firstly, the gold standard involves lifting feet in a restraining chute, we opted to score the feet while they stood in the milking parlor for practical reasons. Secondly, our dataset does not include M1 lesions, as they are not consistently visible in standing cow feet, and M2 lesions are also limited in number.

In the future, we are planning to integrate our app with the other apps such as DD Check app (Tremblay et al., 2016). We can seamlessly detect cow’s lesions and save the details of cows with timestamp so as the veterinarians can identify the cows and treat DD as early as possible. Other possible approaches include image segmentation for more accurate results. But the problem with image segmentation that it is memory intensive and edge devices are not good at handling high memory computation. At present, the model is designed for detecting DD in dairy cows. However, its applicability extends to any other bovine disease exhibiting visible signs. The model, initially trained for dairy cows, can seamlessly be applied to detect diseases in beef cows as well. We have successfully trained the model and obtained preliminary results for the latter scenario. We are also planning to gather more data containing M2-stage lesions.

Monitoring the prevalence of DD at the individual cow level would enhance our comprehension and awareness of DD dynamics within herds endemic to the disease. Currently, numerous instances of DD go unnoticed because the focus of detection is primarily on lame cows displaying acute, active DD lesions (Rodriguez-Lainz et al., 1999). However, this detection strategy overlooks numerous cases of DD, given that not all cows affected by DD exhibit lameness (Tadich et al., 2010).

The use of M-stages has been employed to characterize the clinical progression of DD, highlighting variations in disease severity (Döpfer et al., 1997; Berry et al., 2012). The M-stage system is recognized as a valuable tool in combating DD (Schöpke et al., 2015). It is critical to document signs of chronicity, such as hyperkeratosis and proliferations affected by DD. These factors impact the resulting lameness, infectious potential, and treatment requirements of the DD lesion (Gomez et al., 2015).

The mobile deployment of the YOLOv5 model on an Android and iOS device is not only possible, but very much achievable. Additionally, mobile devices with the upgraded camera and chip specification can provide greater margin of error for distance from object and increased inference time. Mobile applications can be used to monitor the health of individual animals or the entire herd where early detection of health issues can prevent disease outbreaks and improve animal welfare. Real-time data analysis allows for timely decision-making and immediate action to address any emerging issues. Mobile applications allow farmers to remotely monitor their livestock and farm operations from anywhere at any time. This is especially valuable for farmers managing large-scale operations in rural, remote areas. Consequently, object detection apps have the potential to transform disease monitoring and management on dairy farms. The application will enable quick and precise detection of DD lesions, leading to prompt veterinary care and treatment. The ultimate goal is to enhance the welfare and productivity of dairy cattle while reducing the physical pain and economic losses associated with DD and lameness. By deploying object detection models and other CV technology on mobile devices, these applications can impact the welfare of dairy cattle and the production of dairy farming.

## Conclusions

To conclude, we deployed CV models for real-time detection of DD lesions in cows using Android and iOS devices. We applied transfer-learning to DD image data for 5 classes—M0, M2, M2P, M4H, and M4P, on pretrained YOLOv5s model architecture using COCO-128 pretrained weights. During model training, we augmented the data to increase the model robustness in different environments.

The DD models achieved an average mAP of 0.95 on the test dataset. In real-time performance, the agreement reflected by a kappa (McHugh, 2012) score of 0.58 between CV model and the professional annotator is moderate, there remains the potential for significant enhancements. We could leverage our model through the Ultralytics HUB application, that may be optimized for real-time object detection, given that YOLOv5 is developed by the Ultralytics team (Jocher, 2020). Ultralytics has introduced YOLOv8 (Jocher et al., 2023), a new model that promises enhanced performance and flexibility. Alternatively, we could explore other approaches such as employing weighted loss functions, collecting more data and employing data augmentation techniques to address class imbalance (Phan and Yamamoto, 2020).

These mobile apps could be used to perform routine checks of cow’s feet on dairy farms. If signs of DD were seen, farmers could notify veterinarians. The app did not require internet access, making it useful in remote dairy farms with no internet. This type of deployment could be used for any other animal diseases or production outcomes in developing remote regions of the world where web access might be scarce but mobile phones were commonly used. In the future, microcontrollers like Jetson AGX Orin could be used to automate the process and send data directly to veterinarians through the Cloud services like AWS and Google Cloud.
